# How to use communities of practice to support change in learning health systems: A landscape of roles and guidance for management

**DOI:** 10.1002/lrh2.10412

**Published:** 2024-03-05

**Authors:** Stephanie P. Brooks, Esther Ekpe Adewuyi, Tracy Wasylak, Denise Thomson, Sara N. Davison, Kate Storey

**Affiliations:** ^1^ Alberta SPOR SUPPORT Unit—Learning Health System Team, Department of Medicine University of Alberta Edmonton Alberta Canada; ^2^ School of Public Health University of Alberta Edmonton Alberta Canada; ^3^ Strategic Clinical Networks^TM^ Alberta Health Services Edmonton Alberta Canada; ^4^ Faculty of Nursing University of Calgary Calgary Alberta Canada; ^5^ Department of Medicine University of Alberta Edmonton Alberta Canada

**Keywords:** collective case study, communities of practice, implementation, knowledge mobilization, learning health systems, organizational learning

## Abstract

**Background:**

Communities of practice support evidence‐based practice and can be, in and of themselves, applied learning spaces in organizations. However, the variety of ways that communities of practice can support learning health systems are poorly characterized. Furthermore, health system leaders have little guidance on designing and resourcing communities of practice to effectively serve learning health systems.

**Methods:**

We conducted a collective case study, examining a cross‐section of Canadian‐based communities of practice dedicated to supporting evidence‐based practice. We held semi‐structured interviews with 21 participants representing 16 communities of practice and 5 community of practice facilitation platforms that provide administration support, tools, and oversight for multiple communities of practice. Using the Conceptual Framework for Value‐Creating Learning Health Systems, we characterized the numerous roles that communities of practice can take to support learning health systems. We also pulled insights from the interviews on properly resourcing and managing communities of practice.

**Results:**

Communities of practice can advance learning health systems across learning cycles (ie, identifying learning priorities, generating data and knowledge, and implementing and evaluating change). They also act as important infrastructure required to share and coordinate across learning health systems. Community of practice facilitation platforms reduce staff members' workload, in turn, creating greater efficiency and effectiveness across community of practice lifespans. Furthermore, these platforms can be a mechanism to coordinate critical activities (e.g., priority alignment, knowledge brokerage/sharing across the broader system).

**Conclusion:**

To the authors' knowledge, this is the first study to characterize communities of practice across the learning health system landscape. With these results, learning health system leaders have a catalog that clarifies the potential communities of practice roles in knowledge generation, implementation, and uptake of new evidence. Furthermore, the results provide evidence that organizational investment in overarching community of practice facilitation platforms will strengthen and accelerate community of practice supports in learning health systems.

## 1 INTRODUCTION

Communities of Practice (CoPs) are a well‐recognized strategy for deliberately managing and sharing knowledge,^1^ which are also fundamental functions of learning health systems (LHS).^2^ CoPs, simply defined as groups of people who come together to learn from one another, can galvanize knowledge sharing, learning, and innovation, thereby improving organizational performance and competitiveness.^3^, ^4^ Compared to other knowledge management mechanisms, CoPs' strength is their potential to facilitate applied learning environments.^5^ In the health sector, CoPs are employed to facilitate clinical practice improvements and support the implementation of evidence‐based practices.^6^ One challenge for integrating evidence into practice in healthcare is navigating the complex processes of acquiring knowledge and effectively adapting it to fit the context of various clinical settings.^7^ CoPs can ease this challenge as they provide the structure for sharing both explicit knowledge (obtained through training, research, scientific literature, practice guidelines, etc.)^7^ and tacit knowledge (acquired through experiences).^6^

CoPs are traditionally described as emerging, unstructured, and spontaneous.^3^, ^8^ More recently though, research increasingly highlights the role that organizations can play in structuring CoPs.^1^, ^5^, ^9^ Specifically, host organizations can create enabling conditions (e.g., giving clear visions, motivations, systems, and structures) that foster CoP development and sustainment.^4^, ^10^ This ability to enable CoPs will be of particular interest to LHS leaders who oversee improvement and implementation initiatives. A recent systematic review identified that LHS generate measurable health outcome improvements and that CoPs are one of a handful of common infrastructures that contribute to these improvements.^2^ While this evidence is compelling for LHS leaders, the review showed a lack of clarity about CoPs' roles and responsibilities in LHS. For example, in the review, authors discussed four CoP networks, but did not define CoPs or cover how to employ CoPs to strengthen LHS infrastructures and/or processes.^2^ Indeed, in the authors' review of these four CoP networks, only one self‐identified as a CoP.^11^, ^12^ The other networks were described by participants as collaborative research^13^, ^14^ or data‐sharing models,^15^ rather than as CoPs. This discrepancy means it is unclear whether these three other networks were truly CoPs, in the traditional sense, or if these networks were providing other forms of LHS infrastructure.

Health organization leaders need more clarity and guidance to structure and resource CoPs in LHS contexts. In this article, we present interview‐based evidence that supports this clarity. Specifically, we catalog the various ways CoPs can advance LHS and we provide evidence‐based guidance for resourcing CoPs to best support LHS. In this guidance, we argue that investing in a CoP facilitation platform (i.e., an office that facilitates all CoPs in the organization) will strengthen and accelerate CoPs' support of LHS goals.

## 2 RESEARCH QUESTIONS

The specific research questions guiding this study were as follows:

- Where can CoPs contribute to LHS conceptually?
- What resources and/or management do CoPs need to support LHS settings?

## 3 METHODS

We used the collective case study method^16^ paired with the Conceptual Framework for Value‐Creating Learning Health Systems^17^ to (a) develop a landscape of CoP roles within LHS, and (b) identify resources required to effectively use CoPs to advance LHS activities and goals. The collective case study method involves multiple cases that, when analyzed together, generate data about a specific phenomenon.^16^ In this study, cases were Canadian‐based CoPs and CoP facilitation platforms dedicated to supporting evidence‐based practice in health. CoP facilitation platforms managed and facilitated the work of numerous CoPs within a given organization. Boundaries that define cases are essential for setting the scope of case study research, and for distinguishing data from context.^18^ We chose the boundary of evidence‐based practice given its alignment with LHS goals of optimizing care using various forms of evidence.^17^

### 3.1 Conceptual framework used

**FIGURE 1:**
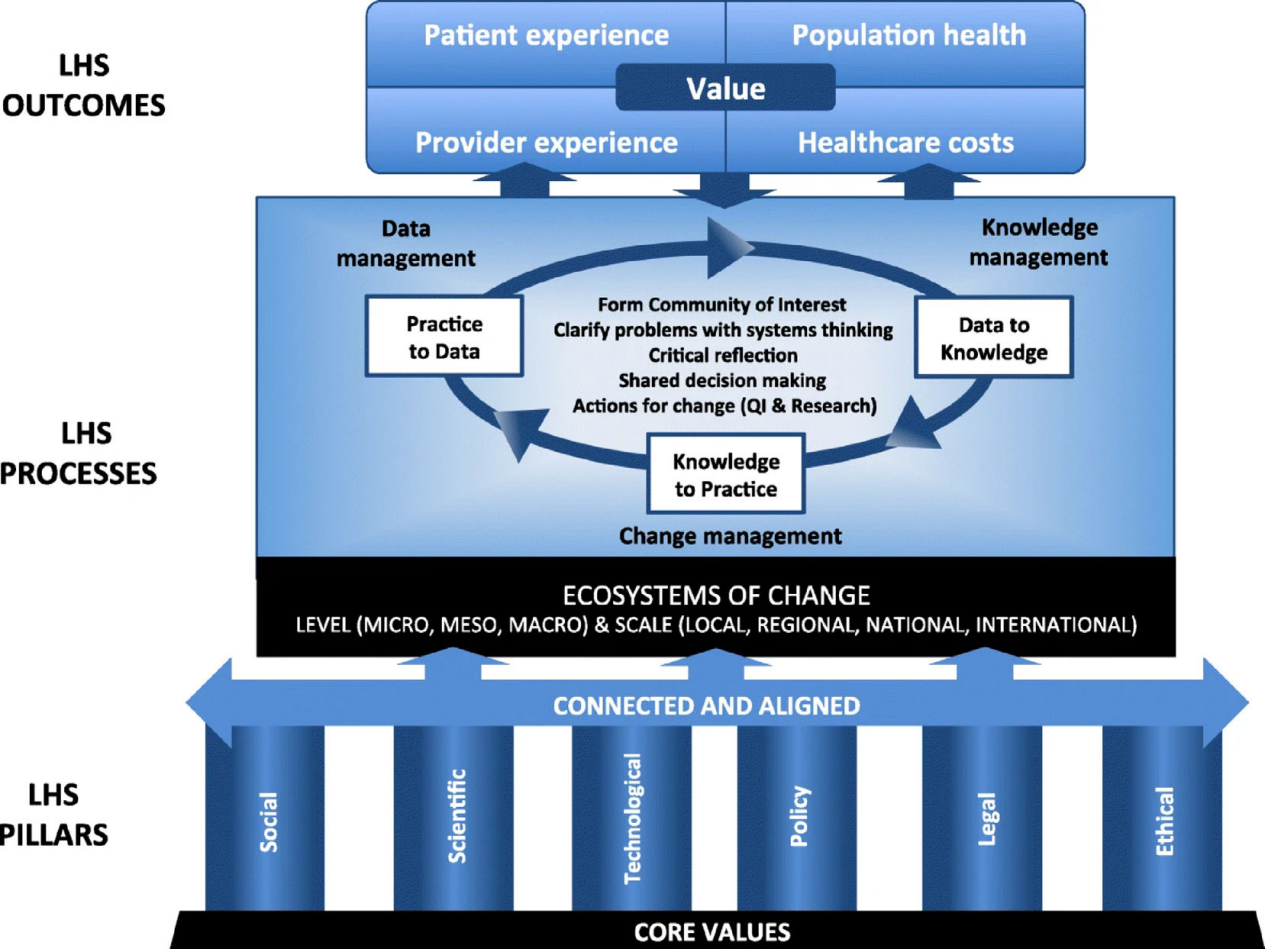
Menear et al.'s Conceptual Framework for Value‐Creating Learning Health Systems^17^ permission to reprint was granted by Springer Nature Group.

To create the CoP in LHS catalog, we mapped our results to the Canadian‐based Conceptual Framework for Value‐Creating Learning Health Systems (Figure 1).^17^ This framework includes the common goals, processes, and infrastructure that make up LHS. We chose this framework to guide our analysis because while Menear et al. built this framework using international LHS evidence, they included insights relevant to universal public healthcare systems, and are therefore relevant to the Canadian context (e.g., values that underpin LHS that would apply to all Canadian health delivery). This differentiates their approach from LHS research carried out in the context of the United States. This alignment between the framework and the context in which our participants work helped us to accurately map interview data to the framework concepts.

Menear et al.'s framework details what conceptual components constitute an LHS and provides examples of each.^17^ The framework details LHS goals, which are aligned with the Quadruple Aim,^19^, ^20^ a common framework used in healthcare that states healthcare organizations should aim to improve patient and provider healthcare experiences, improve patient outcomes, and increase the overall value for money of healthcare delivery. Since this framework was developed, increasing equity in healthcare has been added to the original aims.^21^

LHS continually work towards these goals by conducting LHS processes, commonly called learning cycles.^22^, ^23^ Menear et al.'s framework depicts the three phases of these cycles as Practice‐to‐Data > Data‐to‐Knowledge > Knowledge‐to‐Practice.^17^ In these three‐phase learning processes, (1) data about day‐to‐day care delivery are collected to learn more about provider and user healthcare experiences and health outcomes (i.e., Practice‐to‐Data); (2) data is aggregated and formatted to identify unwarranted variation and inform improvement or innovation decisions (i.e., Data‐to‐Knowledge); and (3) change is planned, implemented, and evaluated to systematically and continuously improve healthcare (i.e., Knowledge‐to‐Practice). Collaboratives called communities of interest initiate and oversee these learning cycles.

The other conceptual categories, LHS pillars and core values represent the people power, infrastructure, and principles required to conduct learning processes. LHS pillars include all infrastructure required to rigorously learn and apply change within an organization. Examples include engagement mechanisms, scientific expertise and training programs, data technologies and systems, as well as legal, policy, and ethical regulations. The numerous values that Menear et al. provide are Canada‐specific and would underpin any functional healthcare organization in the country (e.g., transparency, inclusiveness, shared accountability, and person‐centered). Additionally, Menear et al. identified the importance of effectively connecting and aligning LHS infrastructure and learning cycles to promote system‐wide learning and improvement.^17^

This conceptual framework is tremendously helpful in visualizing the LHS landscape, which includes the purposes, processes, and infrastructure that define LHS. However, the framework does not detail the mechanisms to put LHS processes into motion. For example, Menear et al. mentioned CoPs as an example of social infrastructure; however, they did not explore whether CoPs support other areas of the LHS, which LHS goals CoPs can support, or how to operationalize CoPs. Using Menear et al.'s framework, we mapped the various opportunities to utilize CoPs across the LHS landscape.

### 3.2 Participants

Participating CoPs were identified by searching the Google search engine for “community of practice” and “evidence‐based practice” or “evidence‐informed practice.” We reached out via email to all Canada‐based CoPs identified using website contact forms or contact information provided on the CoP website. Between June and October 2020, we recruited and interviewed 21 participants representing 16 CoPs and five CoP facilitation platforms. All participants were facilitators of their respective CoPs and CoP facilitation platforms.

### 3.3 Data collection

We conducted semi‐structured interviews ranging from 33‐70 min in duration using Zoom video‐conferencing software. We designed the interview guide to learn what practices and resources enable CoPs to effectively support the uptake of evidence‐based practice. The guide included questions about CoP history and purpose, membership, resources and staffing, deliverables, facilitators and barriers to operations, adaptations over time, planning and evaluation practices, and future directions (see Supplement). All questions were asked to all participants with space for the interviewer to probe further into responses. The questions allowed us to collect pragmatic advice around planning, launching, adapting, and sustaining CoPs dedicated to evidence‐based practice.

### 3.4 Data analysis

We used NVivo qualitative management software^24^ to organize and analyze verbatim transcripts of the audio‐recorded interviews. Using the directed qualitative content analysis method,^25^ we examined the potential roles that CoPs can have in LHS contexts and what resources are required to effectively launch and operate LHS‐based CoPs. Directed content analysis requires simultaneously coding transcripts deductively and inductively to test whether data fit into existing models.^26^, ^27^ We deductively coded transcripts using coding categories derived from Menear et al.'s LHS framework (described above)^17^ to identify opportunities to leverage CoPs in LHS. Inductive coding provided insights into important considerations for establishing, repurposing, and/or sustaining CoPs to support LHS. Because we used both inductive and deductive coding, and because we took a constructivist approach, the lead author and another member of the research team (EEA) used a critical friend approach to the data analysis.^28^, ^29^ The critical friend approach allows for exploration of different interpretations of data, in turn checking unconscious bias to entrust rigor in the data analysis. As we completed coding the dataset, no new insights were emerging, indicating data saturation.^30^

Prior to the interviews, all participants in this study provided informed consent to be included in this research. This research design was approved by the University of Alberta Research Information Services, Research Ethics Board I (ID: Pro00100042).

## 4 RESULTS

### 4.1 Structure and function of participating CoPs and CoP facilitation platforms

The participating CoPs and CoP facilitation platforms provided a diverse dataset. The duration of the CoPs and areas of focus ranged widely. CoPs also had a diversity of membership, some serving one type of professional, some serving interprofessional membership, and some serving interdisciplinary members. Furthermore, CoPs varied in strengthening general practice, supporting specific programs, or facilitating research (Table 1).

**TABLE 1:** General characteristics of participating Communities of Practices (CoPs).

Characteristic	CoPs (*n*)
**Area of Interest**	
**Sector**	
Environmental health	1
Palliative care	4
Health administration	1
Knowledge translation	2
**Population**	
Seniors	1
Children	1
**Condition(s)**	
Dementia	1
Infectious diseases	1
Injury prevention	1
Tuberculosis	1
**Treatment**	
Manual therapy	1
Trauma‐informed treatments	1
**Total**	**16**
**Membership Size (*n*)**	
0–100	11
100–1000	1
1000–5000	4
**Total**	**16**
**Membership Type**	
Homogenous	3
Interprofessional	10
Interdisciplinary	3
**Total**	**16**
**Duration (years)**	
<1	1
1–5	10
6‐10	1
10+	4
**Total**	**16**
**Focus of Support** *	
Practice	10
Program‐specific	1
Research	4

* Category counts are non‐exclusive.

Similarly, CoP facilitation platforms each had a sector, population, or condition‐specific focus. Facilitation platforms were responsible for 3‐26 CoPs. While all facilitation platforms were active, the newest platform was two years old and the oldest was fourteen (Table 2).

**TABLE 2:** General characteristics of participating Communities of Practice (CoP) facilitation platforms.

Characteristic	CoP facilitation platforms (*n*)
**Area of Interest**	
**Sector**	
Palliative care	1
Quality improvement	1
Health education	1
**Population**	
Children	1
**Condition(s)**	
Neurological conditions	1
**Total**	**5**
**Currently Active CoPs Supported (** ***n*** **)**	
3	1
5	1
9	1
10	1
26	1
**Total**	**5**
**Duration (years)**	
<1	0
1–5	2
6–10	2
10+	1
**Total**	**5**
**Focus of Support** *	
Practice	3
Program‐specific	3
Research	3

* Category counts are non‐exclusive.

Management and resourcing also varied across the participating CoPs and facilitation platforms, with differing staffing models and funding sources (Table 3). The majority of CoPs were volunteer‐run, where CoP facilitators organized CoP activities outside of their regular work hours and/or on top of their day‐to‐day work duties. Nearly half of the CoPs had at least one paid staff, usually a CoP facilitator responsible for the administrative and/or technological demands of the CoP. In these cases, there was also usually a volunteer or in‐kind CoP lead/director who was responsible for the vision of the CoP but left the execution of CoP activities to the paid staff. Conversely, all but one of the CoP facilitation platforms were staffed entirely by paid staff who were hired to run the platforms. In these cases, the facilitation platforms were responsible for all administrative and technological demands of the different CoPs in their stead. CoP members would volunteer as leaders/content experts and provide vision and direction for the CoP and the facilitation platform would be responsible for execution.

**TABLE 3:** Management characteristics of participating Communities of Practices (CoPs) and CoP facilitation platforms.

Characteristic	CoPs (*n*)	CoP facilitation platforms (*n*)
**Staffing**		
Paid	5	4
Volunteer	9	1
Mixed	2	0
**Total**	**16**	**5**
**Funding Source**		
Grant	6	3
Organization	6	1
Government	2	1
No funding	2	0
**Total**	**16**	**5**
**Evaluations Conducted** *		
Of events/activities	4	3
Of CoP/Platform	4	0
None	8	2
**Total**	**16**	**5**

* Category counts are not mutually exclusive.

The participants described CoP engagement and contribution models (Table 4) that emerged from their various motivations for launching the CoP. Some CoPs were planned to support broader training, program implementation, or research initiatives. The impetuses of other CoPs were reactionary efforts to fill gaps that were keeping people from following evidence‐based practice recommendations (i.e., needing to find knowledge mobilization and knowledge‐sharing mechanisms). At times, these gaps caused patient safety concerns, requiring organizations to find space for interdisciplinary conversations that would improve patient care across care continuums. Some CoP initiation was motivated by a desire to advocate and create best practices for small and dispersed professional groups or region‐specific evidence‐based practice. In these cases, often the CoP would also look to identify exemplary projects or cases to support their advocacy work. Just three of the participating CoPs had an organizational board or steering committee that oversaw their work.

**TABLE 4:** Communities of Practice (CoP) models.

Model: Knowledge mobilization			
Purposes of CoPs	Engagement methods	Contributions	Target CoP members
Promote and support a specific practice model across organizations Support evidence‐based practice for a general area Support knowledge mobilization within one organization Create intersectoral engagement on a condition of interest	Virtual meetings for practice coaching, collaborative tool development In‐person seminars Virtual webinars to present practice advancements and promote new tools Interactive knowledge databases for sharing practice tools and resources (app or website‐based) Online community discussion spaces Library services to provide evidence for practice that is not already publicly available Newsletters Data dashboards	Develop and share tools and resources that would strengthen practice Build individual knowledge mobilization capacity Provide cross‐organizational networking opportunities Provide evidence and experience‐based answers to questions around best practices Provide online or in‐person interdisciplinary collaboration space to develop tools Support working groups to develop and promote policies, strategies and tools related to a condition of interest	Practitioners Researchers Clinicians Cross‐sectoral parties involved in prevention and treatment

Model: Knowledge sharing			
Purposes of CoPs	Engagement methods	Contributions	Target CoP members
Share resources and events about a specific practice, policy, condition, or population	Newsletter Non‐interactive website to house information Evidence database that includes academic literature, grey literature, events, and/or news Moderated website where people can share resources and respond to each other's posts	Create space for members to share research and practice insights with one another Keep community abreast of advances in practice, networking events, and capacity building opportunities	Organizations that support a population of interest Practitioners, researchers, decision‐makers interested in a specific topic

Model: Mandatory participation as part of training, program implementation, or research initiatives			
Purposes of CoPs	Engagement methods	Contributions	Target CoP members
Facilitate groups learning across teams Accelerate development of best practices Capacity‐building for new professionals	Newsletter and/or webinars sharing program or project updates and reports In‐person or virtual retreats Online discussion boards Working groups Training (synchronous and asynchronous) using an online teaching platform	Strengthen work funded by the CoP host Compile and share lessons learned by CoP members (e.g., build reports around research methodologies, implementation strategies, ethical considerations) Increase capacity for a specific practice	Funding recipients Trainees

Model: Interdisciplinary collaboration			
Purposes of CoPs	Engagement methods	Contributions	Target CoP members
Bring researchers across disciplines together to discuss a topic of interest Test and build best practices for a specific research, practice, or policy approach	Meetings (in‐person and virtual) to conduct CoP work Conference attendance, presentations, websites, knowledge databases, public reports to disseminate findings	Share knowledge across CoP members and foster interdisciplinary research collaborations Provide infrastructure for interested teams to identify knowledge gaps, facilitate collaborations to fill gaps, build capacity, and develop best practices	Researchers Researchers and practitioners

### 4.2 LHS‐aligned activities of CoPs and CoP facilitation platforms

Our conceptual mapping of the interview data onto Menear et al.'s LHS framework^17^ provides a landscape of how CoPs and CoP facilitation platforms can support LHS. Specifically, we found that CoPs and CoP facilitation platforms support LHS learning processes and perform social and scientific roles (Table 5). CoP facilitation platforms also serve connecting and alignment functions required in LHS (Table 5). Below, we present the results of LHS concept followed by evidence of adequately resourcing and managing CoPs and CoP facilitation platforms. Each section includes a review of the results and a table with exemplary interview quotes.

**TABLE 5:** Learning health systems(LHS)‐aligned roles of participating Communities of Practice (CoP) and CoP facilitation platforms.

CoPs		
Number of CoPs performing this LHS‐aligned role (*n* *)	CoP purpose(s)	Activities & deliverables
Community of Interest (*n* = 2)	Advocacy for research priorities and/or policy and practice change	Priority identification
Practice‐to‐Data (*n* = 3)	Identify and collect qualitative data to supplement electronic health record data	Research and evaluation frameworks
Data‐to‐Knowledge (*n* = 3)	Intervention research Implementation research Knowledge synthesis Knowledge formatting	Knowledge generation Intervention and implementation evaluation Guideline development Library services and reviews
Knowledge‐to‐Practice (*n* = 9)	Resource sharing and/or training to support evidence‐based practice	Knowledge mobilization Capacity building
Connecting and Aligning (*n* = 1)	Connecting Researchers to Community Organizations	Knowledge mobilization
Social Infrastructure (*n* = 4)	Practice‐based network‐building	Facilitate networking
Scientific Infrastructure (*n* = 1)	Research‐based network‐building	Intervention and implementation research results

CoP facilitation platforms		
Number of CoP facilitation platforms performing this LHS‐aligned role (*n* *)	CoP facilitation platform purpose	Activities & deliverables
Connecting and Aligning (*n* = 1)	Ensure practice changes align with local law and policy	Facilitate KT product and practice guideline development
Social Infrastructure (*n* = 2)	Convene practice and policy‐based CoPs	Support CoPs to identify practice and policy‐based goals/purposes and conduct associated work
Scientific Infrastructure (*n* = 3)	Convene research‐based CoPs Facilitate mentorship	Support CoPs to identify research goals/purposes and conduct associated work

* Counts in categories are not mutually exclusive.

### 4.3 Supporting LHS learning processes

CoPs supported organizational learning in numerous ways. Only one CoP included in this study was explicitly described as an LHS while others performed specific aligned activities. Many CoPs demonstrated supporting learning, in both interprofessional and interdisciplinary ways, often attributing CoP success to having a common purpose or advocacy goal. Often, CoPs performed as communities of interest through their various advocacy work.

The participants described CoPs supporting various learning efforts that spanned the LHS learning cycle (Table 6). Most participating CoPs did not formally collect data or conduct research themselves, though some provided specific data and research support to local service providers. For example, the CoPs following the knowledge mobilization model sometimes took responsibility for building tools and supportive strategies that helped practitioners take up evidence‐based practice. Or these CoPs might provide library services to users who otherwise would not have access to academic evidence. In doing so, CoPs helped identify available data sources, plan programs, and build implementation and evaluation frameworks that allowed teams to collect important quality and implementation outcome data. Furthermore, all CoPs had a knowledge‐sharing function, where members could bring various forms of evidence and discuss how these could inform future practice changes and continuous improvement. This approach to interpreting evidence aligns with the LHS learning cycle's Data‐to‐Knowledge step.

**TABLE 6:** Summary of the potential roles and functions Communities of Practices (CoPs) can play in learning health systems (LHS) learning cycles with exemplar quotes.

Results	Exemplar quotes
*Communities of interest*. Communities of interest emerging from CoPs hinge on advocacy.	*For these communities to work, you really need to identify a topic or an area that is of interest to enough people for it to be successful… for us having a purpose or a goal of the community of practice keeps people coming back because if they can see that if they are coming together, they are having a conversation, but the conversation is moving them towards a solution or towards a product then there is more incentive to continue to meet*. (Platform 3) *Our impetus was really to create an environment and to create a provincial strategy that advocates for the value of [this approach]… we can identify where the exemplar projects are and really understand their behavior, what's happening, and we bring that information back to communities so they can learn through good models and good examples, and to government policy makers and funders to say, “look at the value of these kind of efforts. If we had a little more support or funding, we can sustain these efforts and possibly do even more.”* (CoP 6)
*Practice‐to‐Data*. CoPs can support data collection directly and act as supplementary qualitative data sources.	*We're more interested in identifying some of the unique models that are really capable of changing the delivery structures within communities and doing more population level research…We've been really invested in helping projects collect comprehensive data around those [models], we use the RE‐AIM framework as our program evaluation framework, reach, effectiveness, and maintenance of their work so that we can describe it in our program evaluations… We are now working on doing a much more comprehensive mixed method evaluation form and helping [teams] organize their data so we can measure pre‐ and post‐effectiveness, and so we can do qualitative interviews to understand what the intervention means to different participants in this particular community*. (CoP 6)
*Data‐to‐Knowledge*. CoPs can help to contextualize and enhance electronic health record data.	*We really look at knowledge as being quite broad. It is not strictly research evidence, but we will often include environmental scans of what other initiatives or projects that are going on, research itself, other literature. We'll certainly look at what's the practice‐based knowledge and marry those. Then we really try to incorporate lived experience too, depending on what is the work that we're trying to do, but often we will incorporate the lived experience whether that's people living with [the condition of interest], care partners, or others, whoever has lived experience of what it is we're trying to accomplish. Then marrying those so that it creates new knowledge in that process. Then thinking about that translation of how we structure that knowledge in a way that it's going to be usable and that we can put into action*. (Platform 1)
*Knowledge‐to‐Practice*. CoPs can be employed to plan and optimize change.	*We would like [healthcare delivery teams] to come back to us and say, “You're connected with the researchers, could you connect us with a researcher or let them know that in the trenches where we're trying to apply the research, this is working, and this is not.” We look for that bidirectional flow*. (CoP 1) *We find that when groups have a very specific purpose for coming together and have a very specific target audience then there seems to be more engagement and more success in running a CoP. Versus being a more general CoP that's just open to everyone…When you bring people together a very specific group of people together for a very specific purpose it's part of their job, a need to do, vs a nice to do. That's where there is a high engagement because people have questions, they have challenges, it's part of their role, it's something that they need to get done from A to Z, and there is a high level of engagement*. (Platform 4)

CoPs supported change in several ways that would support and strengthen the Knowledge‐to‐Practice step of the LHS learning cycle. For example, one CoP organized itself to let healthcare teams discuss their experiences implementing an evidence‐based practice or intervention and troubleshoot barriers they faced. Half of the participating CoPs supported change through more traditional knowledge (e.g., increasing access to new knowledge). One of the facilitation platforms had overseen nearly 70 CoPs over their tenure and reflected that using CoPs to directly support the implementation of change created the strongest engagement by CoP members. Consequently, engagement helped to strengthen evidence‐based practices.

### 4.4 CoPs as LHS social and scientific infrastructure

CoPs provided important infrastructure for the social requirements of LHS (Table 7). Regardless of the overall purpose or goal of each individual CoP, their work brought people together who would otherwise remain unconnected. Furthermore, participants emphasized that the nature of CoPs makes them natural mechanisms for collaboration across organizations, sectors, and geographies. This collaboration was strongest when there was a work‐related purpose for the CoP, rather than attending CoPs out of general interest in the topic area or field. Some participants described members naturally breaking off from the first CoP to start new ones with narrower, but more job‐specific foci.

**TABLE 7:** Summary of how Communities of Practices (CoPs) provide social and scientific learning health systems (LHS) infrastructures with exemplar quotes.

Results	Exemplar Quotes
*Social Infrastructure*. Engagement appears to increase when people are brought together around a specific piece of work.	*When you bring a very specific group of people together for a very specific purpose, it's part of their job, a need‐to‐do, vs a nice‐to‐do. That's why there is high engagement, because people have questions, they have challenges, it's part of their role, it's something that they need to get done from A to Z, and so there is a high level of engagement… You need to be part of this group in order to get your work done*. (Platform 4) *The purpose [of this CoP] was inter‐disciplinary, was to bring together people with diverse background but also diverse skills and artistic genres being represented. What started happening was that people who were working in research‐based theatre started splintering off. People who were working with music‐based things started splintering off. They started forming their own research interest group, which was great but then it was really difficult to bring people together in that more diverse group*. (CoP 13)
*Scientific Infrastructure*. CoPs can also bring scientific expertise together to support program delivery.	*The research CoP is made up of academics and the delivery CoP is made up for program people. The researchers will come from time to time to different delivery CoP meetings, and there is an open invitation for those on the delivery side to identify their own projects and come discuss those ideas with the research CoP… Sometimes teams will have their researchers join the research CoP and the project lead join the delivery CoP so that's good when that happens… there is lots of cross pollination and certainly, all of the information is available to both. But they are different groups of people with different areas of expertise and different priorities in terms of what they're trying to accomplish in their respective networks*. (CoP 7)

The social nature of CoPs also supports the scientific infrastructure required of LHS. A minority of the CoPs conducted research, but most focused on exchanging knowledge, with some specifically dedicated to discussing various research methods (Table 5). The CoP established to be an LHS was made up of two separate CoPs that worked together to perform as one LHS. One CoP represented delivery and implementation, while the other was responsible for research. This model allowed delivery CoP members to discuss and refine various care models prior to implementation. Meanwhile, the research CoP supported monitoring and evaluating the different models using implementation science to identify the potential scalability and sustainment of various approaches (Table 7).

### 4.5 CoP facilitation platforms perform LHS aligning functions and strengthen CoPs

While participants described individual CoP roles in learning, CoP facilitation platforms were described as important connectors between CoPs and other individuals or groups across the system (Table 8). In addition, these platforms ensured CoP work aligned with the interests and priorities of the host organization or other key partners. Moreover, these facilitation platforms performed administrative functions that help individual CoPs avoid common pitfalls, such as being managed “off the side of a staff member's desk”. This administrative support spanned CoP lifespans from initiating the CoPs to sunsetting. For example, CoP facilitation platforms reduced front‐end work by shepherding CoPs through all of the considerations involved with conceptualizing, launching, and operating CoPs. Participants also described how CoP facilitation platforms were particularly valuable for bringing specialized skills that supported CoP knowledge synthesis and mobilization activities. Trained facilitators were also valuable CoP facilitation platform members as they carefully managed group dynamics and supported time‐sensitive or multisectoral work. More generally, facilitators created space for members to actively engage at CoP meetings.

**TABLE 8:** Summary of how Communities of Practice (CoP) facilitation platforms support the learning health systems (LHS) and the individual CoPs.

Results	Exemplar Quote
*Connecting and Aligning*. CoP facilitation platforms have an important bird's eye view of different teams, activities, and stakeholders.	*It makes it easier to solve problems, or to further an action or an ask to different levels of government, if we are all focused on something together. We do take into account what our partners' and stakeholders' priorities are as well. So that we're not working against each other, we're working with each other. That's really an important part of our strategy as well. Really our value is that you're working in partnership with other people and other organizations*. (Platform 3)
*Administration*. CoP facilitation platforms eliminate the risks associated with managing CoPs informally.	*If you have somebody who's more dedicated to [administration], and actually had a chunk of time that they were supposed to use to lead this community of practice, it would really lead to more success*. (CoP 11) *There is so much potential with them, but we're limited by money and time. that is a challenge, giving it the time it needs to make it successful otherwise it's just a black hole. If we had a full‐time person other more urgent things wouldn't have to take priority. We used to have CoP support on retainer and [the CoP] was better with that support than it is now*. (CoP 9) *Our process really helped them. When we were talking to a group, we were orienting them, and they really understood their own role and how they could contribute. And I think that's what's missing when people come together to create a community of practice. The lines between these things are so blurred that people want to participate but they may not be very clear on how and how long it's going to take, and how can they be most successful in a community… When someone comes to me and says, “I want to create a community of practice,” this is part of my knowledge translation, this is where I say okay let me give you a little bit of community of practice 101*. (Platform 4) *Our staff group always does the facilitation of the meetings. You need that meeting structure and need the facilitation. We're just trying to make sure the facilitation creates open dialogue space*. (Platform 2) *But you need defined leadership, and you need subject matter experts who can help keep things moving forward. As a staff member who is not a subject matter expert, I can keep things moving forward administratively and with engagement, with communication, with all of those things I can do that but I'm not the subject matter expert so I need you to tell me what exactly it is we're doing. So really success relied on having individuals sponsored by the organization to ensure the work is done*. (Platform 3)
*Facilitation and Acceleration*. CoP facilitation platforms strengthen and accelerate CoP activities, especially if they have knowledge management skills.	*We currently have 26 groups, we have sunsetted over 50. Groups come on, they come off, they come on, they come off. Sometimes they come on for six months, sometimes they come on for two years, whatever time, and then they sunset. We now have people who are least familiar with [the platform] and they come and use it in different ways. They might go to the posts page, they may join another community of practice, or they may want to create their own community of practice once they've already been a member of a group. You have this word of mouth that kind of happens and familiarity and awareness building when people have been a part of a group on [our platform]*. (Platform 4) *I was there as the research methodologist because I have a background in design, research, and methodology… My task was to try to keep [the CoP members] on the methodology instead of getting only involved in the clinical aspects, because they were supposed to be learning critical appraisal and research design. It was very easy for them to get off on their clinical area of expertise otherwise*… *The librarian was a big draw… When we could get myself, a clinician, the librarian, and someone else, it was great*. (CoP 2) *[Our CoP facilitation platform] used to provide a knowledge broker to help several CoPs. That made it possible for us to be able to do a lot of good work. We were working more actively on tools. We're working on a new tool now and started on it last year, but it's hard going on with it without access to a knowledge broker*. (CoP 4)
*Responsiveness*. CoP facilitation platforms helped create new CoP or re‐orient existing CoP activities to respond to emergent needs (e.g., issues that emerged from the COVID‐19 pandemic).	*It just depends on the time of the year, which subgroups are being used, and what the roles are for even just the CoP members. But this year has just been particularly different, just with everything that's going on and just with COVID and things like that. So, the role has been very dynamic*. (CoP 10) *We are happy to provide the support to faculty who are interested in exploring things that are either relevant, because of the timeliness or particular events that happen, or just because it's important to them…I'm saying there is just an opportunity to do things that have not been done in the past. If there is interest, we respond to that*. (Platform 5)
*Resourcing*. CoP facilitation platforms require ample resourcing and personnel to support the various CoPs across the system.	*Management and leadership support within our organization is essential to remove barriers, discuss any challenges*, etc. *You need a CoP manager, someone in the driver's seat. I will always say when someone is starting a community of practice if you don't have someone with dedicated time and support to run that community of practice from a very administrative, operations level the car will not go forward. I've mentioned if you don't have champions and experts there to support you and to advise you and to help you with the work from more content expertise, you are not going to be successful*. (Platform 4)

Beyond day‐to‐day support, CoP facilitation platforms helped their organizations respond to emergent needs. Many of the participants said that the COVID‐19 pandemic hindered or halted CoP operations. Conversely, others had the opposite experience, where they rapidly re‐evaluated their purpose to meet the needs emerging from the pandemic. CoP facilitation platforms described helping CoPs reframe their purpose when needed. All CoP facilitation platforms emphasized that to benefit CoPs, they needed organizational leadership and sufficient investment (e.g., dedicated time for administrative duties, a champion to motivate participation, funding, leadership support, etc.).

## 5 DISCUSSION

Our results clarify where CoPs can support LHS and how to intentionally employ CoPs to strengthen LHS functions. With these results, health system leaders have a catalog of the roles that CoPs can play in knowledge generation, implementation, and uptake of new evidence in LHS. Specifically, our results show that CoPs can (a) act as communities of interest, (b) strengthen learning cycles, (c) provide social and scientific infrastructure, and (d) play important roles in coordinating and aligning work between teams across a given LHS. Furthermore, the results provide evidence that organizational investment in an overarching CoP facilitation platform will strengthen and accelerate CoP supports. CoP facilitation platforms can help CoPs avoid front‐end meeting and committee fatigue that can slow change initiatives. They also help CoPs meet emergent and ongoing needs in LHS.

### 5.1 CoP roles in LHS

As highlighted by other research, CoPs clearly belong in LHS, either as infrastructure^17^ or as spaces for learning.^2^, ^31^ Furthermore, CoPs have been shown to support LHS at various system levels (i.e., micro, meso, and macro levels).^5^ While not conducted in LHS settings, other studies illustrate that CoPs can perform important LHS functions, such as issue clarification,^5^ systems thinking,^1^, ^32^ critical reflection,^1^, ^31^, ^32^ shared decision making,^33^, ^34^ and identifying actions for change.^35^ This knowledge base makes a strong case for the utility of CoPs in LHS; however, previous studies have failed to provide clarity around exactly what LHS roles CoPs can perform. This study brings forth this required clarity, demonstrating that CoPs can specifically strengthen learning and knowledge application in LHS contexts.

Our study substantiates beliefs that CoPs can support transdisciplinary collaboration,^33^, ^36^ a defining activity of LHS.^17^ Participants in our study emphasized that the nature of CoPs makes them mechanisms for collaboration across organizations, sectors, and geographies. The participants in this study described CoPs that served homogenous members (e.g., specialists), interprofessional members (eg, different professions involved in a given care pathway), or interdisciplinary membership, (e.g., patients, practitioners, and designers who work together to build physical environment interventions). Thus, the CoP structure has desirable flexibility that allows LHS leaders to utilize CoPs to respond to emergent LHS needs. They can build capacity in specific target groups or they can support implementation of initiatives that involve multiple teams and disciplines. This ability to traverse and break down silos is a highly desirable and unique feature of LHS compared to traditional health organizations.^37^ This finding illustrates CoPs' suitability to become LHS communities of interest (i.e., transdisciplinary teams that identify needs for learning health cycles and also oversee these cycles).

Beyond the connecting and oversight functions that CoPs can play, our study shows that CoPs can also support learning throughout the LHS learning cycle phases. Indeed, one participating CoP described itself as an LHS model, made up of two complementary CoPs, one practice and policy‐oriented and the other research‐oriented. These CoPs worked together to identify and tackle various questions to strengthen clinical practice and improve patient outcomes. This is an example of how CoPs can support the LHS learning cycle holistically. Many of the other participating CoPs showed how they support specific phases of LHS learning cycles instead. Specifically, our findings uncovered examples of CoPs helping to (a) collect practice‐based data, including qualitative data collection, that enhanced administrative data; (b) interpret data during analysis; and (c) support implementation and change management. Traditionally, CoPs are often initiated to provide networking opportunities and spaces for sharing knowledge and resources.^33^ Our results suggest that the strongest CoPs are developed to help people complete specific pieces of work. This is important for LHS leaders to consider when looking to intentionally employ and resource CoPs to support change.

### 5.2 Organizational use of the results

Establishing CoPs with intentionality can maximize CoP impact and overcome CoP pitfalls.^33^ Intentionality fosters strategic relevance and alignment with organizational goals, two critical elements of CoP success.^6^, ^34^ CoPs in LHS contexts will perform best when intentionally established to transcend practice, organizational, and sectoral silos by facilitating cross‐sectoral learning.^12^ The results of this study provide health organization leaders with a catalog of CoP functions, activities, and potential contributions to LHS. LHS leaders can use this knowledge to inform how to set up CoPs in LHS with more intentionality. Our study results can help leaders think through which research and change initiatives would benefit from CoPs, how to recruit sufficient membership for the desired change, and how to manage CoPs to achieve LHS goals. For example, LHS leaders can use the insights from the mandatory CoP model to inform push approaches to change, such as setting up a CoP to accelerate program implementation or uptake of best practices. Conversely, our data about knowledge translation and resource sharing CoP models suggest that LHS leaders can also use existing CoPs as mechanisms to inform pull approaches to change. These CoPs can be used to gain insights on capacity‐building or infrastructures required for staff at various levels of the organization to perform their work in evidence‐based ways.

Finally, the results from our study show that overarching CoP facilitation platforms strengthen organizations' ability to intentionally employ CoPs by acting as a potential conduit between LHS leadership and CoPs. Overarching CoP facilitation platforms strengthen organizations' ability to intentionally employ CoPs. These platforms also reduce CoP administrative burden, subsequently allowing them to focus on the work that will strengthen the LHS. Furthermore, CoP facilitation platforms perform critical LHS connecting and aligning functions.

### 5.3 Benefits of investing in a CoP facilitation platform

Our findings show that the belief that CoPs require little to no resources is a fallacy. Growing evidence shows that CoP work is often run “off the side of the desk” and is not valued or maintained the same as other more urgent daily work, despite the benefits that CoPs bring to organizations.^5^ Adequate resourcing for launching and maintaining CoP activities is critical for CoP success.^6^, ^34^ Our results show that these resources include paid personnel with dedicated time for CoP coordination and management. These personnel include CoP leaders responsible for the vision of the CoP, administrative support, and technical support for CoPs that utilize virtual meeting spaces or other technical communication and work infrastructure. Given that all CoPs will require these resources, LHS leaders can consider investing in a CoP facilitation platform that can move individual CoPs through the growing pains involved with conceptualization, launch, and operations. Our participants also hailed the meeting facilitation support that these platforms can provide. As our findings highlighted, this support reduces administrative burden, helping CoPs work more efficiently and effectively. Moreover, establishing CoPs through a platform enables systematic documentation of CoP implementation. Few CoPs report using a framework or systematic approach to planning and operations.^32^ If CoP facilitation platforms used a standard framework in their work, the LHS could use an implementation science approach to learn which CoP models, implemented in which ways, work for different contexts.

On top of administrative expertise, our results showed that having knowledge synthesis and mobilization expertise increased CoPs facilitation platforms' value. CoP facilitation platforms can include research and evidence services that will accelerate LHS learning cycles. When CoP facilitation platforms provide knowledge management and mobilization skills, they strengthen and accelerate the use of knowledge created within CoPs. These staff could include librarians, knowledge brokers, statisticians, data scientists, and implementation scientists who can synthesize knowledge, format it for use, and facilitate change planning and evaluation.

The centralized nature of CoP platforms can enhance the connecting and aligning functions of individual CoPs. With an overarching view of all CoP activities and a staff with evidence support expertise, CoP facilitation platforms can also serve alignment and coordination functions for the broader LHS (e.g., identify cross‐sectoral issues, find collaboration opportunities, and align CoP activities with organizational goals). Our results showed that existing CoP facilitation platforms already do this, as evidenced by their assistance in helping CoPs respond to emergent needs during the COVID‐19 pandemic. These findings suggest that CoP facilitation platforms could contribute to LHS responsiveness.

### 5.4 Future research

Our study is an initial step towards defining and cataloging CoPs within LHS. Our results showed how CoPs can be employed to collect and analyze qualitative data that complements electronic health record data. Future studies are needed to identify other CoP‐supported synergies, such as assessing whether CoPs can help design easy‐to‐use electronic health record data collection and actionable data use mechanisms (ie, data systems that inspire change). Impact assessments and cost‐benefit analyses of CoPs and CoP facilitation platforms would help to refine our characterization of CoPs in LHS, identify what models work best for different goals, and what specific resources are required for different models. This knowledge would bolster other evidence showing that CoPs can help to implement and sustain innovations.^36^ To achieve this level of detailed knowledge of CoPs in LHS, more evaluations of CoP impact are required.^38^, ^39^ While this study begins to bring clarity to CoPs as one approach for bringing people together around goals that support LHS, future research is needed to define and characterize other approaches (e.g., integrated learning collaboratives, research and practice networks, etc.).

## 6 STRENGTHS AND LIMITATIONS

This study included CoPs and CoP facilitation platforms from different health sectors, disciplines, and geographies across Canada, which increased the generalizability of our findings. This approach allowed us to see patterns across sectors, matching LHS goals of enabling transdisciplinary work. Despite the diversity of our overall sampling, we only spoke with active CoPs. Consequently, we did not learn about critical conditions or resourcing required to avoid CoPs from becoming defunct. Furthermore, we only collected data from the CoP perspective, not from CoP host organizations. Thus, our results cannot inform best practices for CoPs from the perspective of the organization. Our data was also sensitive to recall bias and weak institutional knowledge, as some of the participants were new CoP facilitators or were not privy to decisions made about the CoPs or facilitation platforms before they came into the facilitator role. Our data generation strategy allowed us to develop a descriptive understanding of CoPs in LHS but could not provide any explanatory power to this study. Therefore, we could not provide a determinant model or framework to be considered best practice for those looking to design and resource CoPs. This limitation is compounded by the innovative nature of CoPs. Over time, new facilitation techniques, engagement platforms, etc., will emerge, meaning similar analyses will need to occur periodically to update our knowledge of CoP resource needs and practices. Participants were identified through websites, creating a bias towards lessons learned from publicly facing CoPs only. Lastly, most of the participating CoPs and facilitation platforms were not part of an existing LHS, and thus, could not comment on their role in LHS specifically.

## 7 CONCLUSION

CoPs have become commonplace in health settings, but CoPs remain under‐characterized in LHS literature, making it difficult for health organization leaders to know when and how to best utilize CoPs. CoPs can strengthen health organizations' ability to respond to emergent issues. This responsiveness is a key feature of LHS.^17^, ^23^ This study provides evidence on how to employ CoPs to meet ongoing and emergent issues in LHS contexts. This work to characterize CoPs in LHS is one step towards identifying best practices for using CoPs in these areas. A key point of clarity brought from this study is that CoPs are labor‐intensive, thus the costs of maintaining CoPs must be balanced with other health system needs. Our evidence suggests that investing in CoP facilitation platforms provides an overarching infrastructure that can reduce the overall CoP administrative burden while accelerating and strengthening work completed by CoPs.

## CONFLICT OF INTEREST STATEMENT

The authors of this article have no conflicts of interest to declare.

## Supporting information


**Data S1.** Supporting Information.
