# Supplementary material for: How to use communities of practice to support change in learning health systems: A landscape of roles and guidance for management
Source: Learn Health Syst. 2024 Mar 5;8(3):e10412. doi: 10.1002/lrh2.10412 (PMC11257050; doi:10.1002/lrh2.10412)
Supplement: Supplementary file 1 — Data S1. Supporting Information. [file LRH2-8-e10412-s001.docx]

Thank you very much for agreeing to participate in this study. The SPOR KT Platform appreciates you taking the time to share your experiences in launching, operating, or using [insert community of practice name]. Your insights from [planning, running, using] this community of practice are very valuable to us. Please feel free to share any ideas or reflections you have from your time with community of practice name] that you think would help others planning a similar community of practice in their local contexts.

1. **Can you describe your role with [community of practice name]? Please be specific about the nature and level of your involvement.**
2. **Can you please tell me about the [community of practice name]? Its' purpose, size, the members it serves, etc.**

[PROBING QUESTIONS]

1. What are the primary goals?

2. Who does the community of practice serve and why did you focus on that community?

3. Has the size of membership changed? If so, to what do you attribute the change?

4. How do you connect with members and deliver content? Why did you choose this(these) method(s)?

5. Have any of these characteristics changed over time? If so, to what do you attribute these changes?

1. **Can you please give us a little history of the community of practice? Where did the idea to create this come from and how did you get it off the ground?**

[PROBING QUESTIONS]

1. What resources did you have to plan, launch and operate this community of practice?

2. Did you consult with anyone or do any research before launching? If so, can you elaborate? Did you find that valuable? Are there resources you would recommend to others?

3. What kind of partners, if any, were involved in designing the community of practice and how did they contribute?

1. **What other roles, paid or volunteer, are necessary to keep [community of practice name] running?**

[PROBING QUESTIONS]

1. Personnel-wise, what manpower doing which tasks are required to keep this community of practice running day-to-day?

2. What are all the tasks required for [community of practice name] to function as intended? How many people does it take to cover all of these tasks?

3. What other resources does this community of practice require to function as intended?

1. **How do you/your team go about ongoing planning for the community of practice?**

[PROBING QUESTIONS]

1. Do you meet regularly to discuss planning, activity delivery, sustainability, etc? If so how often and what are the typical agenda items for these meetings? If not, please describe how you make decisions about the community of practice’s upcoming activities, growth, and sustainability.

2. What informs your planning (e.g. evaluation, surveying members, etc).

1. **What are some of the activities, deliverables or outputs you create and deliver? Why did you choose these types of things to focus on?**

[PROBING QUESTIONS]

1. Are your activities and resources intended to promote evidence to be used, to guide how to implement evidence into practice, other things? Are these publicly available?

2. Which activities or resources are more useful and/or impactful for your members? Why do you think this is the case?

3. Have the activities or resources you deliver changed over time? If so, how so and why did you make these changes?

1. **Do you perform evaluations?**

[PROBING QUESTIONS]

1. If yes, how frequently and what measures do you use to evaluate success?

1. **What has contributed to the success of the community of practice?**

[PROBING QUESTIONS]

1. Of the goals you mentioned earlier, which have you achieved and which are you still working towards?

2. What conditions exist that have facilitated/are facilitating learning and change?

1. **What have been some challenges of launching and/or operating [community of practice name]? What did you do to overcome these challenges?**

[PROBING QUESTIONS]

1. Which of these challenges were expected and which were unexpected?

2. How did you prepare for the expected challenges?

3. How did you respond to unexpected challenges?

1. **From your experience, what are some of the most important considerations, activities, or milestones that people need to think about in planning, launching, and operating a community of practice like yours?**
2. **What additional advice would you give to other groups trying to launch a similar community of practice to promote or support evidence-based practice?**
